# microRNA-20a in human faeces as a non-invasive biomarker for colorectal cancer

**DOI:** 10.18632/oncotarget.6403

**Published:** 2015-11-26

**Authors:** Tung On Yau, Chung Wah Wu, Ceen-Ming Tang, Yingxuan Chen, Jingyuan Fang, Yujuan Dong, Qiaoyi Liang, Simon Siu Man Ng, Francis Ka Leung Chan, Joseph Jao Yiu Sung, Jun Yu

**Affiliations:** ^1^ Institute of Digestive Disease and Department of Medicine and Therapeutics, State Key Laboratory of Digestive Disease, Li Ka Shing Institute of Health Sciences, CUHK Shenzhen Research Institute, The Chinese University of Hong Kong, Hong Kong; ^2^ Oxford University Clinical Academic Graduate School, John Radcliffe Hospital, Oxford, UK; ^3^ Renji Hospital, Shanghai Jiaotong University, Shanghai, China; ^4^ Department of Surgery, The Chinese University of Hong Kong, Hong Kong

**Keywords:** microRNA, non-invasive, stool biomarker, colorectal cancer, diagnosis

## Abstract

**Objective:**

Detection of microRNA (miRNA) aberrations in human faeces is a new approach for colorectal cancer (CRC) screening. The aim of this study was to characterise miR-20a in faeces as a non-invasive biomarker for diagnosis of CRC.

**Results:**

miR-20a expression was significantly higher in the 40 CRC tumours compared to their respective adjacent normal tissues (*P* = 0.0065). Levels of miR-20a were also significantly higher in faecal samples from CRC patients (*P* < 0.0001). The area under receiver operating characteristic (AUROC) curve for miR-20a was 0.73, with a sensitivity of 55% and specificity of 82% for CRC patients compared with controls. No significant difference in the level of miR-20a was found between patients with proximal, distal, and rectal cancer. The use of antibiotics did not influence faecal miR-20a levels.

**Patients and Methods:**

miR-20a was selected from an expression microarray containing 667 miRNAs. Further verification of miR-20a was performed in 40 pairs of primary CRC tissues, as well as 595 faecal samples (198 CRCs, 199 adenomas, and 198 healthy controls) using TaqMan probe based quantitative Real-Time PCR (qRT-PCR).

**Conclusions:**

Faecal-based miR-20a can be utilised as a potential non-invasive biomarker for CRC screening.

## INTRODUCTION

Colorectal cancer (CRC) is the third most common cancer worldwide, with incidence rates increasing by 6% over the past decade [1]. CRC typically develops from benign adenomas to malignant adenocarcinomas through a long and protracted stepwise process. Patient survival is inversely related to the cancer stage at diagnosis, with up to 90% of deaths preventable if diagnosed early [2]. However, colorectal cancer is frequently asymptomatic in its early stages. Hence, the development of non-invasive biomarkers for screening the populations at risk is urgently needed [3].

miRNAs belong to a class of highly conserved short single-stranded non-coding RNAs, which regulates messenger RNA (mRNA) degradation, and inhibits translation of target genes via binding to the 3′-untranslated regions (3′UTR). Since miRNA expression profiles between normal and tumour cells, as well as between different subtypes of cancers vary due to their unique clinical histopathologic features, miRNAs are ideal cancer biomarkers [4]. miR-20a belongs to the miR-17/92 cluster located in the 13q31.1 region, and is up-regulated in numerous cancers, including anaplastic thyroid [5], ovarian [6], and prostate cancer [7, 8]. Notably, this area is partly regulated by the oncogenic transcription factor Myc [9] and TGF-β [10]. Over-expression of the miR-17/92 cluster is thus associated with accelerated cell proliferation [11], tumour development [12], and transformation from benign adenomas to CRC [13].

Data from our miRNA microarray, which was previously reported [14], demonstrated that miR-20a was the one of most up-regulated miRNA in tumours compared to adjacent normal tissues. Thus, the purpose of this study was to evaluate the expression of miR-20a in faeces as a non-invasive CRC diagnostic biomarker. We began by using 40 paired clinical CRC tissues to validate miR-20a expression. Next, miR-20a expression was validated in faecal samples from a large cohort of 595 patients, including 198 with CRC, 199 with adenomas, and 198 healthy controls. Through this large case-controlled study, we identified and characterised faecal-based miR-20a as a potential non-invasive biomarker for CRC diagnosis.

## RESULTS

### miR-20a is significantly up-regulated in primary CRC compared to their adjacent normal tissues

Amongst the 667 miRNAs we screened using a microarray reported previously [14], miR-20a was the most up-regulated miRNA in tumour specimens compared to its adjacent normal. Thus, miR-20a was selected for further validation in 40 paired tumour and corresponding adjacent normal tissues from CRC patients. We found that miR-20a expression was significantly up-regulated (fold change: 2.063 (0.910–5.418), *P* = 0.0065) in tumours compared to adjacent normal tissues (Table 1).

**Table 1:** miR-20a expression is elevated in colorectal carcinoma tissues compared with adjacent normal tissues

microRNA	Percentage of samples with elevated expression in tumours	Fold change (Interquartile range)	*P* value
miR-20a	70.0% (28/40)	2.063 (0.910–5.418)	0.0065

### Faecal-based miR-20a is a potential non-invasive marker for colorectal cancer

**Figure 1:**
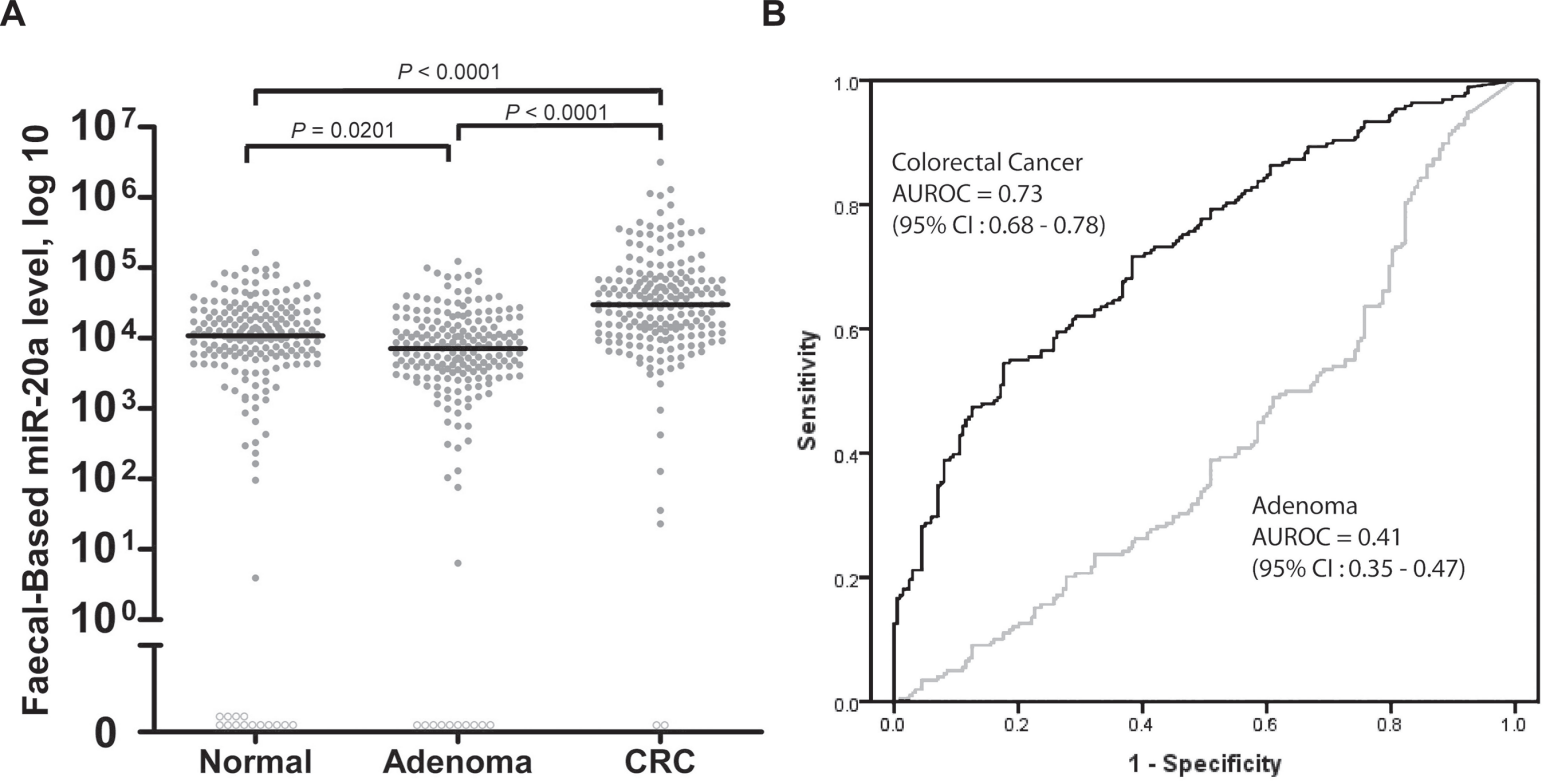
Levels of (A) faecal-based miR-20a, and (B) the respective area under receiver operating characteristic (AUROC) curves for CRC and adenoma Patients were categorised into three subgroups: individuals with a normal colonoscopy (normal) (*n* = 198), adenoma (*n* = 199), and CRC (*n* = 198). The miR-20a level was expressed as the number of copies per nanogram of extracted total RNA. Each open circle represents a sample with an undetectable miRNA level. The lines denote the median. *P* < 0.05 denotes statistical significance. AUROC curves were plotted to discriminate all CRC and adenoma patients from individuals with normal colonoscopy findings.

miR-20a was evaluated in three groups of participants, that is groups with normal colonoscopy (*n* = 198), adenoma (*n* = 199), and CRC (*n* = 198) (Table 2). As shown in Figure 1A, miR-20a was able to discriminate between patients with CRC and healthy individuals. Statistically, faecal-based miR-20a levels were significantly higher in CRC (mean: 100,827 copies/ng, 95% confidence interval (CI): 114,870–86,783 copies/ng; median: 30,005 copies/ng; *P* < 0.0001), but also significantly lower in adenoma (mean: 13,199 copies/ng, 95% CI: 15,033–11,365 copies/ng; median: 7,088 copies/ng; *P* = 0.0201) compared to controls (mean: 18,051 copies/ng, 95% CI: 20,566–15,537 copies/ng; median: 10,776 copies/ng) (Figure 1A).

**Table 2:** Pathological characteristics of recruited subjects

Category	Healthy Controls	Adenoma	Colorectal Cancer
No. of Cases	198	199	198
Age at enrolment, Years (Mean ± SD)	58.65 ± 6.87	59.99 ± 5.97	66.53 ± 11.05
**Gender, Number (%)**			
Male	84 (42%)	114 (57%)	116 (59%)
Female	114 (58%)	85 (43%)	82 (41%)
Location*, Number (%)			
Proximal			50 (25.3%)
Distal			82 (41.4%)
Rectum			66 (33.3%)
**Cancer stage, Number (%)**			
I + II			106 (53.5%)
III + IV			88 (44.5%)
Unknown			4 (2.0%)
**Tumour histology, Number (%)**			
Adenocarcinoma			185 (93.4%)
Mucinous adenocarcinoma			11(5.6%)
Unknown			2 (1.0%)
**Differentiation, Number (%)**			
Poor			1 (0.5%)
Poor to Moderate			2 (1.0%)
Moderate			167 (84.3%)
Well to Moderate			3 (1.5%)
Well			3 (1.5%)
Unknown/No data			22 (11.2%)
Antibiotic intake**, Number (%)			
Yes			26 (13%)
No			172 (87%)

* Colorectal neoplasms were classified by location into three groups: proximal colon (caecum, ascending, hepatic flexure and transverse), distal colon (splenic flexure, descending and sigmoid and recto-sigmoid junction) and rectum.

** Antibiotic intake is defined as any antibiotic intake in the 30 days preceding faecal sample collection.

The AUROC values of faecal-based miR-20a were 0.73 (95% CI: 0.68–0.78) in CRC, and 0.41 (95% CI: 0.35–0.47) in adenoma (Figure 1B). The cut-off value of 27,493 copies/ng of extracted total faecal RNA for miR-20a was selected to maximise the sum of the sensitivity and specificity for CRC diagnosis (Table 3). miR-20a had a sensitivity of 55% and specificity of 82% for CRC detection. The second cut-off value of 43,312 copies/ng for miR-20a (Table 3) was chosen for its high specificity of 90% enabling assessment of its performance for reference.

**Table 3:** The sensitivity and specificity of faecal-based miR-20a for colorectal cancer detection

Category	Best	Reference
**Specificity, % (95% CI)**	82 (76–87)	90 (85–94)
**Sensitivity, % (95% CI)**	55 (47–62)	40 (33–47)
**Cut-off value, copies/nanogram**	27,493	43,312
Location*, Sensitivity % (95% CI)		
Proximal	42 (28–57)	30 (20–45)
Distal	60 (48–70)	45 (34–57)
Rectum	58 (45–70)	45 (33–58)
Antibiotic Intake**, Sensitivity % (95% CI)		
No	27 (10–40)	8 (3–14)
Yes	50 (30–70)	42 (23–63)

* Colorectal neoplasms were classified by location into three groups: proximal colon (caecum, ascending, hepatic flexure and transverse), distal colon (splenic flexure, descending and sigmoid and recto-sigmoid junction) and rectum.

** Antibiotic intake is defined as any antibiotic intake in the 30 days preceding faecal sample collection.

Faecal-based miR-20a in combination with our previously reported faecal miRNA biomarkers miR-92a [15] or miR-135b [14] did not show a big improvement in sensitivity. When miR-20a is combined with miR-92a, the AUROC is 0.77 (95% CI: 0.72–0.82), with a sensitivity and specificity of 57% and 84% for CRC, respectively. If combined with miR-135b, it generates an AUROC of 0.79 (95% CI: 0.74–0.83), with a sensitivity and specificity of 79% and 65% for CRC, respectively (Supplementary Figure S1).

### Faecal-based miR-20a is not associated with the location of CRC

**Figure 2:**
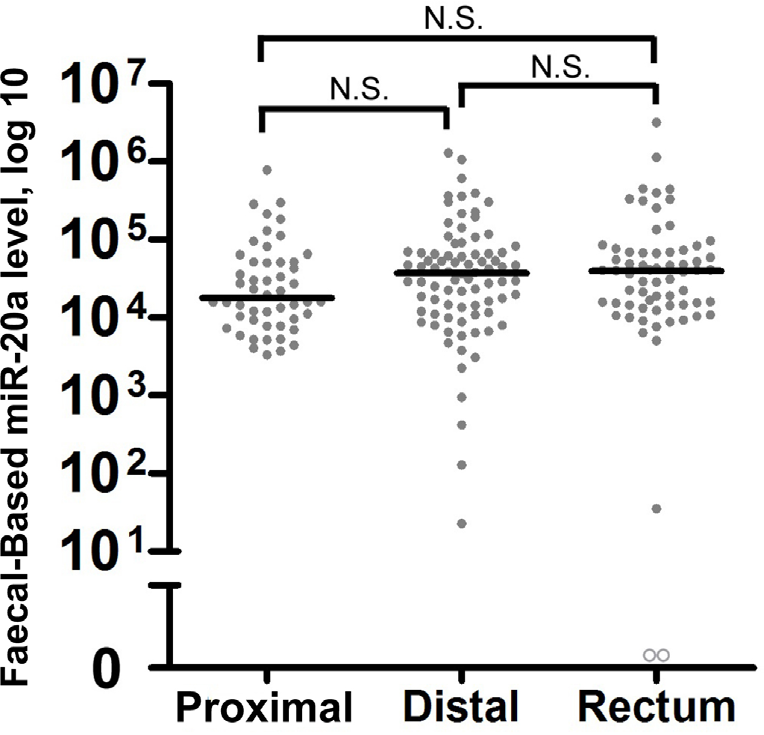
Tumour location does not significantly alter faecal miR-20a levels Colorectal neoplasms were classified by three locations as follows: the proximal colon (caecum, ascending, hepatic flexure and transverse) (*n* = 29), distal colon (splenic flexure, descending and sigmoid and recto-sigmoid junction) (*n* = 75), and rectum (*n* = 66). The lines denote the median. N.S. denotes no statistical significance. miR-20a levels were expressed in number of copies per nanogram of extracted total RNA. Each open circle represents a sample with an undetectable miR-20a level.

We evaluated the expression levels of faecal-based miR-20a in the context of tumour location in CRC patients. No significant differences were observed with regards to sensitivity for the detection of CRCs from the proximal colon, distal colon, and rectum (Figure 2).

### Faecal-based miR-20a expression is not associated with antibiotic intake

**Figure 3:**
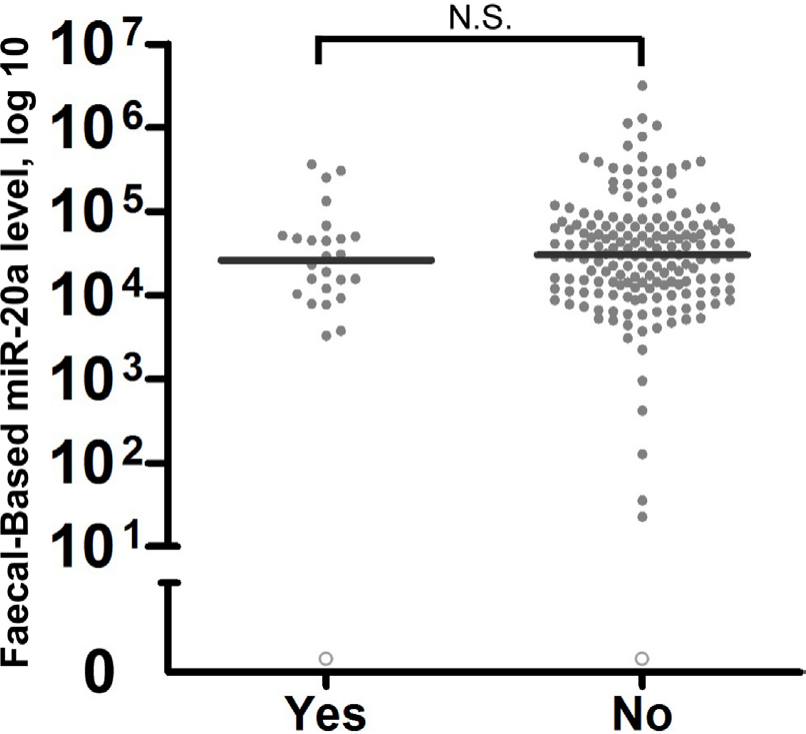
Evaluation of the effects of antibiotics on faecal-based biomarker miR-20a Patients who took antibiotics within 30 days of specimen collection (*n* = 26) were compared with patients without any antibiotic intake (*n* = 162). The lines denote the median. N.S. denotes no statistical significance. miR-20a levels were expressed in number of copies per nanogram of extracted total RNA. Each open circle represents a sample with an undetectable miR-20a level.

We investigated the effects of antibiotic intake on faecal miR-20a. Twenty-six CRC patients had taken antibiotics within one month of faecal collection, whereas the remaining 162 CRC patients had not. There were no significant differences in faecal-based miR-20a expression between the groups with or without antibiotic intake (Figure 3).

## DISCUSSION

CRC is associated with a highly recognisable, and homogenous pattern of miRNA alterations in human faeces [16]. miRNA in faeces is also stable in room temperature and in a 4°C refrigerator for up to 72 hours, with the results from faecal samples being highly repeatable [15, 17, 18]. Unlike the faecal occult blood test (FOBT), which is currently used for CRC screening, faecal-based miRNA tests do not require troublesome drug and dietary restrictions. Therefore, the uptake of faecal-based miRNA tests may be higher than that of the FOBT, which currently stands at 35% [19]. As a result, quantitation of miRNA biomarkers in human faeces by qRT-PCR is a promising non-invasive approach for screening CRC patients [14, 15, 20, 21]. We have previously investigated the expression profile of 667 mRNAs in a microarray, and reported miR-20a as a potential biomarker [14, 21].

Its potential as a biomarker is supported by various functional studies implicating miR-20a in tumourigenesis. miR-20a has been found to induce epithelial-mesenchymal transition (EMT) – a key step in cell migration and tumour metastasis-via down-regulation of E-cadherin, and up-regulation of matrix metalloproteinases [22, 23]. miR-20a has also been shown to diminish cellular response to the TGF-β signalling pathway by preventing its delay of G1/S transition and promoting progression into the cell cycle [10, 22]. Mutational inactivation of the TGF-β signalling pathway is critical in CRC progression, with restoration of the TGF-β pathway in human CRC cells abrogating proliferation and tumourigenicity [24]. Collectively, these functional studies suggested a role for miR-20a in the pathogenesis of CRC, and supported the use of miR-20a as a non-invasive biomarker.

In this study, we began by verifying miR-20a expression levels in 40 paired tissues from CRC patients. miR-20a was confirmed to be more highly expressed in tumours than in their adjacent normal tissues (Table 2). Next, we quantitated miR-20a in human faecal samples from 595 subjects, including 198 patients with CRC, 199 patients with adenoma, and 198 individuals with a normal colonoscopy (Table 1). miR-20a was significantly increased in CRC patients (*p* < 0.0001, AUROC = 0.73) compared with the control group (Figure 1). No difference was found between different genders (Supplementary Figure S2A), and early stage (stages I + II) versus late stage (stages III + IV) CRC patients (Supplementary Figure S2B). Studies by other groups have also demonstrated that faecal miR-20a expression was significantly lower after curative CRC surgery, highlighting a potential role for miR-20a in surveillance of CRC recurrence [25]. Collectively, this data demonstrates the ability of miR-20a to differentiate patients with CRC from those without, supporting its use in CRC diagnostics.

Rather unexpectedly, miR-20a expression levels were lower in adenoma than in healthy controls (*p* = 0.0201, AUROC = 0.41) (Figure 1). A review of the literature revealed no published studies on faecal miR-20a expression in patients with colorectal adenomas. One study reported tissue miR-20a expression in colorectal adenomas, and found that expression was higher in paraffin-embedded colorectal adenoma samples (*n* = 7) than healthy controls (*n* = 9). The difference, however, was not significant, and the small sample size made the findings unreliable [26]. We hypothesise that the lower expression levels are instead due to the influence of the gut microbiome on miRNA within host cells [27, 28]. This hypothesis is supported by recent studies which revealed the different, and unique microbiota profiles of healthy patients, patients with colorectal adenomas, and patients with CRC [29]. The dominant strains of bacteria in colorectal adenomas may degrade miR-20a in the bowel lumen, thus reducing miRNA expression in faecal samples. It is also known that over time, gut flora may alter gene expression in colonocytes [30, 31]. This may also result in lower expression levels of faecal miR-20a in colorectal adenoma patients. Further research is needed to evaluate the relationship between the gut flora and expression of miR-20a in patients with colorectal adenomas.

We also investigated external factors which may affect the use of miR-20a as a faecal-based biomarker. We found that miR-20a levels have comparable efficacy for the detection of proximal colon, distal colon and rectal CRC. Whilst levels of faecal-based miR-20a were slightly lower in proximal CRC than distal and rectal CRC, this result was not statistically significant (Figure 2). Other research groups have demonstrated that antibiotics change the composition of intestinal microbiota, which may in turn alter miRNA expression in faeces [32]. Therefore, we also looked into the effects of antibiotics on faecal-based miR-20a levels by comparing patients who took antibiotics within 30 days of the faecal sample collection and those who had not. There were no significant differences in faecal-based miR-20a expressions between the groups (Figure 3). However, further studies are needed to determine the effects of antibiotics on the faecal-based miRNAs reported by other groups. Nevertheless, this evidence is significant since antibiotic use is common. Thus restrictions to antibiotic use prior to testing for miR-20a are not required to optimise test performance.

Thus far, our results suggest that faecal-based miR-20a is a potential non-invasive biomarker for CRC detection. Opponents, however, argue that faecal-based miRNA tests face similar challenges to the faecal occult blood test (FOBT) in terms of low patient acceptability. Other groups have thus investigated the use of circulating miR-20a in CRC diagnosis. The majority of studies found that circulating miR-20a was unable to differentiate CRC patients from healthy controls in a statistically significant manner [33, 34]. Only one study, in a cohort of 100 CRC and 79 cancer-free controls, reported a statistically significant difference (*P* = 0.038). However, it had a low AUROC of 0.59, with a sensitivity of 46%, and specificity of 73% [35], making it an ineffective diagnostic tool. Moreover, the levels of circulating miR-20a may be influenced by other factors, including chronic diseases such as HCV-mediated liver disease [36], systemic lupus erythematosus [37] and chronic obstructive pulmonary disease (COPD) [38], as well as other malignancies [39–46]. We believe that the higher specificity of faecal miR-20a makes it a better choice for CRC diagnosis than circulating miRNAs.

Nevertheless, there were several shortcomings with our study. Several internal control genes such as 18S rRNA [47], endogenous control small RNAs (i.e. RNU19 [18] and U6 snRNA [48]), miR-16 [25], and miR-24 [49], were used in other faecal-based miRNA studies to determine the relative miRNA levels according to the 2(−ΔΔCt) method. However, recent research has suggested that the use of internal controls for faecal-based miRNA detection may not be an ideal approach [15, 18]. This is firstly because 18S rRNA, RNU19, and U6 snRNA have longer sequences and degrade rapidly in faeces, thus potentially confounding results [15]. As the function of miR-16 and miR-24 itself are unknown [49], there may also be unintended repercussions to using it as an internal control. In our experiment, miRNA was quantified with a standard curve plotted by known amounts of synthetic miRNA and normalised to per nanogram of input RNA. Whilst this overcomes the faults of using internal control genes, this approach may also be problematic because the standard curve is only as good as the quantification method and does not eliminate the possibility of DNA contamination. Our laboratory is currently working on solutions to this problem using multiplex PCR, as well as digital droplet PCR (ddPCR) to optimise performance, and to increase the sensitivity and specificity. Using multiplex PCR techniques, which facilitates detection of multiple targets in a single PCR reaction, our previously reported faecal miRNA biomarkers [14, 15, 21] can be combined with miR-20a in a panel to increase its overall sensitivity and specificity. Likewise, published studies suggest the use of ddPCR, which enables absolute quantification, would increase test performance by reducing the coefficient of variation by up to 86% compared to qRT-PCR [50]. Collectively, detection cost, time, and consumables would be minimised, whilst maximising test performance.

In summary, our study demonstrated that faecal-based miR-20a can be utilised as a potential non-invasive biological marker. Its use in combination with previously reported miRNA biomarkers can be an effective way of screening the population for CRC in a non-invasive manner.

## PATIENTS AND METHODS

### Tissue and faecal sample collection

Forty pairs of primary CRC and its adjacent normal tissues (at least 40 mm away from the tumour margin) were biopsied during the initial colonoscopy or the surgical resection. The specimens were snap frozen immediately in a liquid nitrogen filled vacuum flask, and transferred to a −80°C freezer for storage.

Faecal samples were collected using a 30 mL disposable container with a screw cap from 595 subjects (198 CRCs, 199 adenomas, and 198 neoplasm-free controls) (Table 1). The containers were manufactured under aseptic conditions to minimise the possibility of contamination. Faecal samples from CRC patients were collected 7 days after colonoscopy, whereas samples from adenoma and control groups were collected before bowel purgation and colonoscopy. All faecal samples were stored immediately at 4°C following collection, and transferred to a −80°C freezer for storage within 24 hours.

Colorectal neoplasms were categorised by three locations as follows: the proximal colon (caecum, ascending, hepatic flexure, and transverse), distal colon (splenic flexure, descending, sigmoid, and recto-sigmoid junction), and rectum. Exclusion criteria included: (i) patients who were passing type 7 stool on the Bristol stool chart [51], (ii) previous adjuvant therapy and/or colonic surgery for CRC, as well as (iii) subjects with a family history of familial hereditary non-polyposis CRC and/or familial adenomatous polyposis. All participants had signed informed consent for obtaining tissue and/or faecal samples, and were recruited from The Prince of Wales Hospital, The Chinese University of Hong Kong, Hong Kong and The Alice Ho Miu Ling Nethersole Hospital, Tai Po, Hong Kong. The institutional review board of the Hospital Authority of Hong Kong and the Chinese University of Hong Kong approved of the study protocol.

### MicroRNA extraction in tissue and faecal samples

Frozen colorectal tissue (10–20 μg) from biopsies were added into 500 μL of Trizol reagent (Invitrogen, Carlsbad, CA, USA) in a 1.5 mL RNase free micro-centrifuge tube. The tissue was homogenised by RNase-free pestles and vortexed for 30 seconds to allow for complete homogenisation. 100 μL of chloroform was subsequently added to the 1.5 mL tube. Faeces (200–300 mg) were scooped from the container, and added into 1 mL of Trizol LS reagent (Invitrogen, Carlsbad, CA, USA) in a 2 mL RNase-free microcentrifuge tube (Invitrogen, Carlsbad, CA, USA). The faecal sample was subsequently deformed by a RNase-free pestle (USA Scientific, Woodland, CA, USA) and homogenised by a vortex mixer in the Trizol LS reagent. After completing the homogenisation, 200 μL of chloroform was added into the 2 mL microcentrifuge tube.

Total RNA, including miRNA from tissue and faeces, were extracted from the Trizol-chloroform and Trizol LS-chloroform mixture respectively using the miRNeasy Mini Kit (Qiagen, Valencia, CA, USA) as per the protocols provided. Total RNA was eluted in 50 μL of nuclease free water. Total RNA concentration was measured using the Nanodrop 2000 (Thermo Fisher Scientific, Waltham, MA, USA). Each total RNA sample was normalised to 2 ng/uL based on the Nanodrop 2000 reading.

### MicroRNA quantitation by quantitative real-time PCR

Reverse transcription was performed using the TaqMan miRNA Reverse Transcription Kit (Thermo Fisher Scientific, Waltham, MA, USA). In brief, 2 ng total RNA, 0.3 μL TaqMan miRNA RT primer, 3 nM dNTP (with dTTP), 10 units reverse transcriptase, 0.6 units RNase inhibitor, and 0.3 μL 10X RT buffer were used in one RT reaction with a total volume of 3 μL. The thermal cycling conditions were as follows: 16°C for 30 minutes, 42°C for 30 minutes, 85°C for 5 minutes, and hold at 4°C. The RT product was subsequently diluted four-fold by adding 9 μL of nuclease free water.

qRT-PCR of miR-20a was carried out using the TaqMan has-miR-20a Assay (Assay ID: 000580; Mature sequence: UAAAGUGCUUAUAGUGCAGGUAG) (Thermo Fisher Scientific, Waltham, MA, USA), and the 7500 real-time PCR system (Thermo Fisher Scientific, Waltham, MA, USA). The PCR reaction mix contained 10 μL 2X TaqMan Universal PCR Master Mix with no AmpErase Uracil N-Glycosylase (UNG), 0.5 μL miRNA TaqMan primers, 4 μL diluted RT product, and 5.5 μL nuclease free water. The PCR profile was as follows: 95°C for 10 minutes, 50 cycles of 95°C for 15 seconds, and 60°C for 1 minute. Data collection was carried out at each 60°C step. The quantitation of miR-20a was based on a standard curve plotted by known input amongst all of the miRNAs, and normalised to per nanogram of the total input RNA. Based on standard curves plotted from known amounts of synthetic miR-20a, a technical detection limit of 6 copies for miR-20a would give an approximate Ct value of 48. Consequently, we assigned “0” to all Ct values larger than 48 for miR-20a. Samples with no amplification of miR-20a were also included and assigned a value of “0” in the analysis, provided the sample could be amplified by another miRNA such as miR-135b [14], miR-221, or miR-18a [21]. All assays were performed in a blinded fashion.

### Statistics

The difference between miRNA expression in paired CRC and adjacent normal tissue specimens was evaluated by the Wilcoxon matched-pairs test. AUROC curves were generated based on faecal miRNA levels in patients with CRC and adenoma compared to the control group. Differences in faecal miRNA levels between groups were analysed by the Mann Whitney *U* test. The best cut-off value, selected to maximise the sum of the sensitivity and specificity, and a cut-off with a high specificity of 90%, were selected using the AUROC curve for CRC. A *p* value < 0.05 was considered statistically significant. The AUROC analysis was done by SPSS 16.0 (SPSS Inc., Chicago, Illinois, USA). All other statistical tests were performed using GraphPad Prism 5.01 (GraphPad Software Inc., San Diego, CA, USA).

## SUPPLEMENTARY FIGURES AND TABLES


