# Supplementary material for: microRNA-20a in human faeces as a non-invasive biomarker for colorectal cancer
Source: Oncotarget. 2015 Nov 26;7(2):1559–68. doi: 10.18632/oncotarget.6403 (PMC4811480; doi:10.18632/oncotarget.6403)
Supplement: Supplementary file 1 [file oncotarget-07-1559-s001.pdf]

# microRNA-20a in human faeces as a non-invasive biomarker for colorectal cancer

## Supplementary Materials

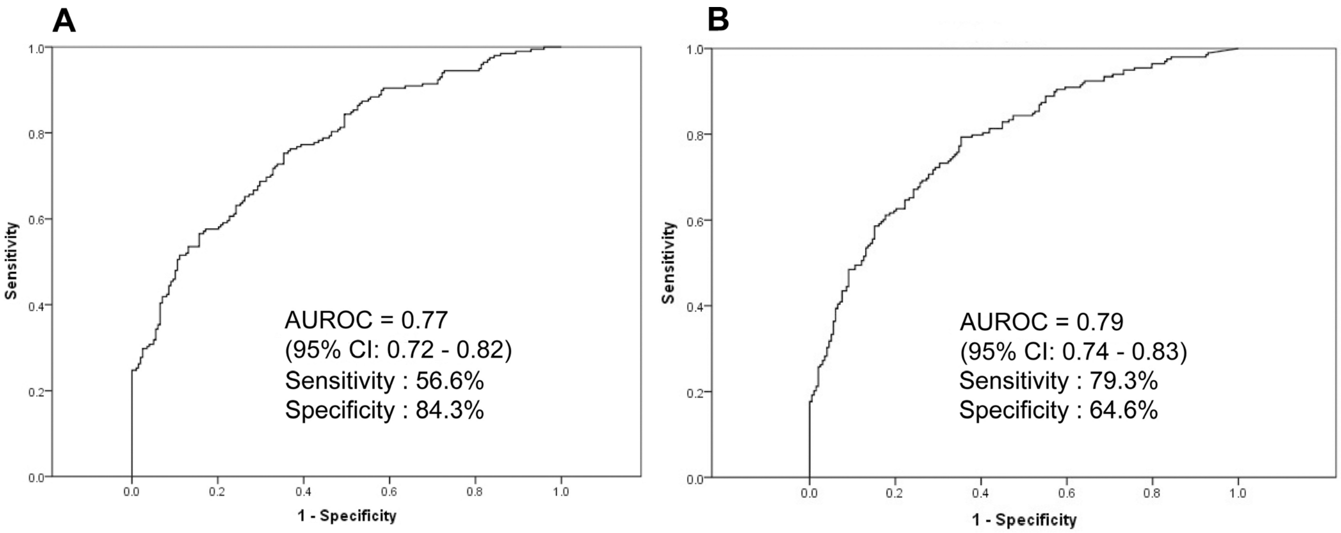


**Supplementary Figure S1: AUROC curves for miR-20a in combination with (A) miR-92a or (B) miR-135b.** The miR-20a level was expressed as the number of copies per nanogram of extracted total RNA. AUROC curves were plotted to discriminate all CRC patients from individuals with normal colonoscopy findings.

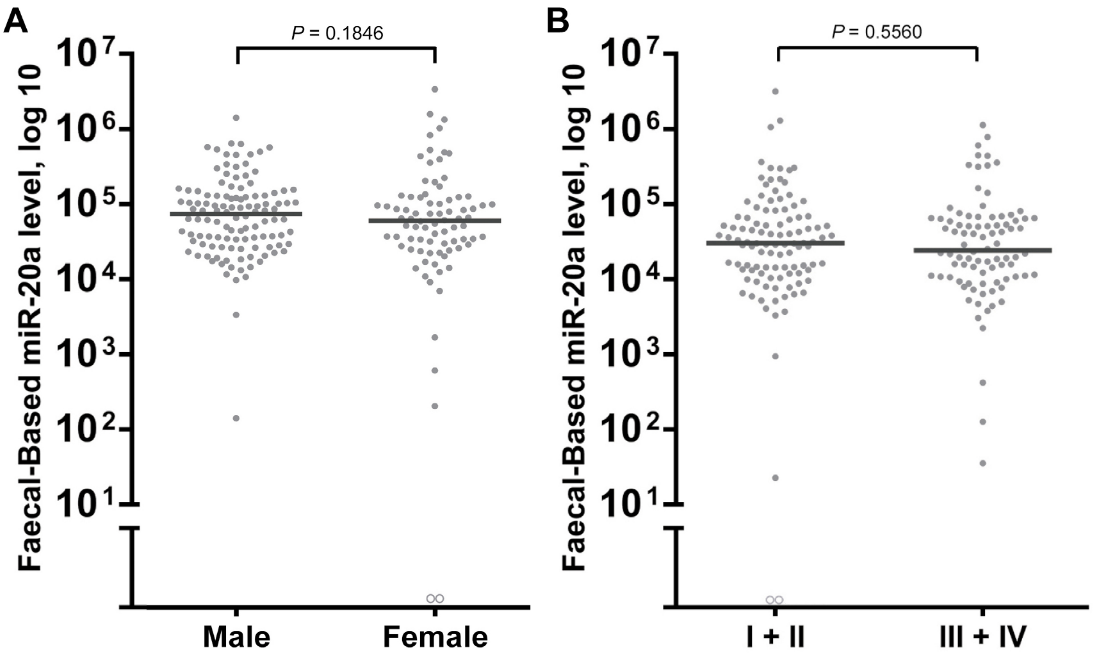


**Supplementary Figure S2: Levels of faecal-based miR-20a levels in CRC patients ($n$ = 198).** There are no statistically significant differences between (**A**) male ($n$ = 116) versus female ($n$ = 82) patients ($P$ = 0.1846) and (**B**) early stage (I + II) ($n$ = 106) versus late stage (III + IV) ($n$ = 88) patients ($P$ = 0.5560). The lines denote the median. N.S. denotes no statistical significance. miR-20a levels were expressed in number of copies per nanogram of extracted total RNA. Each open circle represents a sample with an undetectable miR-20a level.
